# Three Siblings With a Rare Familial Hyperphosphatemia Syndrome: A Case Series

**DOI:** 10.7759/cureus.55575

**Published:** 2024-03-05

**Authors:** Zaid A Sowaity, Jaber Y Saleem, Tayseer N Sabooh, Osama N Dukmak, Sima Y Abu Al-Saoud

**Affiliations:** 1 Faculty of Medicine, Al-Quds University, Jerusalem, PSE; 2 Department of Pediatrics, Makassed Hospital, Al-Quds University, Jerusalem, PSE

**Keywords:** tumoral calcinosis, hyperphosphatemia, galnt3 mutation, hhs, htfc

## Abstract

Hyperphosphatemia familial tumoral calcinosis (HFTC) and hyperphosphatemia hyperostosis syndrome (HHS) are rare autosomal recessive disorders caused by mutations in the polypeptide N-acetylgalactosaminyltransferase 3 (GALNT3), fibroblast growth factor 23 (FGF23), or klotho (KL) genes. They are characterized by hyperphosphatemia and recurrent episodes of bone lesions with hyperostosis and/or soft tissue calcinosis. Management options include phosphate-lowering therapies, anti-inflammatory medications, and surgical excision of the calcified masses in significantly disabled cases. We describe three cases from a consanguineous family who were found to have the same genetic mutation caused by a homozygous mutation in intron eight of GALNT3 c.1524+1 G>A (IVS8+1). The first case had a presentation similar to chronic osteomyelitis, while the second one presented with a calcified mass in her gluteal area. The third case presented with left leg pain. Being a rare disease, the findings of tumoral calcinosis/ bony abnormalities, along with elevated phosphate levels, should raise the possibility of this entity. Family history and biochemical findings can help reach the diagnosis.

## Introduction

Hyperphosphatemia familial tumoral calcinosis (HFTC) is a rare metabolic disorder with less than 100 genetically confirmed cases reported in the literature [1]. It is characterized by an increase in the level of phosphate in the blood and abnormal calcium-phosphate crystal deposition in soft tissues like skin, muscles, and joints [2,3].

HFTC and hyperphosphatemia hyperostosis syndrome (HHS) were thought of as two different entities with different etiologies, but it has later been shown that they are different manifestations of the same genetic defect [4-6]. HHS is characterized by calcinosis in the bone, whereas HFTC is characterized by calcinosis in extraosseous tissue [7].

The disease is caused by a mutation in the polypeptide N-acetylgalactosaminyltransferase 3 (GALNT3), fibroblast growth factor 23 (FGF23), or klotho (KL) genes. The FGF23 gene encodes a protein called fibroblast growth factor 23, which decreases circulating phosphate levels by downregulation of sodium phosphate cotransporter, the major phosphate transporter in the renal proximal tubule, and by downregulation of 25-hydroxyvitamin D-1-alpha hydroxylase. The GALNT3 gene encodes an enzyme called UDP-N-acetyl-alpha D-galactosamine, which protects intact FGF23 from catabolism and inactivation post-translational glycosylation. Therefore, this disorder can be either secondary to an inactivating mutation in GALNT3 preventing proper a-linked glycosylation of FGF23 or due to a mutation in the FGF23 gene. As a result, the mutations will lead to increased renal tubular phosphate reabsorption and usually elevated 1,25-dihydroxy vitamin D3, which promotes gastrointestinal absorption of calcium and phosphate [8]. As for the role of the KL gene, studies have shown that klotho is an additional cofactor that is required by FGF23 to exert its activity, as it converts canonical FGF receptors into specific receptors for FGF23, thus allowing their binding and signaling through the receptors [9].

We present three Palestinian siblings of consanguineous parents with variable presentations of HFTC/ HHS.

## Case presentation

Case 1

A 13-year-old male presented to our hospital with left leg mid-shaft pain for one month. The patient's history dates back to the age of seven years. He had a history of recurrent episodes of right lower leg pain, for which he underwent extensive investigations. CT showed right proximal tibial metaphyseal-diaphyseal bone marrow infiltration with periosteal reaction suggestive of an inflammatory process. Assuming osteomyelitis, he was treated with multiple courses of intravenous antibiotics and underwent debridement and curettage.

Upon presentation to our hospital laboratory investigations, as described in Table 1, showed high serum phosphate 7.95 mg/dl (normal range: 2.5-4.5), with normal calcium, parathyroid hormone, alkaline phosphatase, renal function tests, complete blood count (CBC), and inflammatory markers (C-reactive protein, CRP, erythrocyte sedimentation rate, ESR). X-ray of the left lower leg showed a periosteal reaction. A whole-body MRI showed diffuse intra-medullary altered signal involvement of the left tibia, with similar findings in the right femur and left proximal humorous. Bone biopsy from the left tibia showed fragments of woven bone, bone marrow spaces, and occasional necrotic bone spicules surrounded by a chronic inflammatory cell infiltrate.

**Table 1:** Main laboratory tests of the patients CRP - C-reactive protein; ESR - erythrocyte sedimentation rate; BUN - blood urea nitrogen; LDH -  lactate dehydrogenase; TRP - tubular reabsorption of phosphorus; TSH - thyroid-stimulating hormone

Laboratory tests	Case 1	Case 2	Case 3	Normal range	Unit
White blood cells	8.62	5.79	10.1	4.60-10.2	×10˄9/L
Hemoglobin	12.5	12	12.2	12.2-18.1	g/dl
Platelet	346	462	303	142-424	×10˄9/L
Neutrophils	48.2	37.5	42.6	37-80	%
ESR	6	15	9	0-20	mm/hr
CRP	3	4.5	0.3	0-6	mg/L
BUN	9	10.06	7.8	6-20	mg/dL
Creatinine	0.56	0.56	0.51	0.5-0.9	mg/dL
Albumin	4.3	4.8	4.7	4.1-4.8	g/dL
Phosphorus	7.95	8.45	7.75	2.5-4.5	mg/dL
Calcium	10.2	10.06	10.2	8.6-10.2	mg/dL
Potassium	4.4	4.46	4.02	3.5-5.5	mEq/L
Magnesium	1.8	1.9	1.7	1.6-2.6	mg/dL
LDH	146	200	150	135-225	U/L
Alkaline phosphatase	156.4	139	152.3	40-480	U/L
Parathyroid hormone	15.8	23	24	9-90	pg/ml
25-OH vitamin D	17.1	10.8	13.5	20-42	ng/ml
TRP	98%	96%	99%	82-95	%
TSH	1.2	1.31	1.2	0.4-4	mIU/L
Free T4	1.5	1.4	1.4	0.5-2	ng/dl
Uric acid	5.17	3.7	3.5	3.4-7	mg/dL
Creatine phosphokinase	70	136	100	0-170	U/L

Based on the imaging and lab findings of hyperphosphatemia, HHS was suspected. The diagnosis was confirmed by genetic testing for mutations in the GALNT3 and FGF23 genes. The patient was found to be homozygous for the mutation c.1524+1 G>A (IVS 8+1) in intron eight of the GALNT3 gene. 

He was started on a low phosphorus diet, phosphate binder agent (sevelamer carbonate), and was given nonsteroidal anti-inflammatory drugs (NSAIDs) as needed. Later, aluminum hydroxide was added due to persistent hyperphosphatemia.

**Figure 1:**
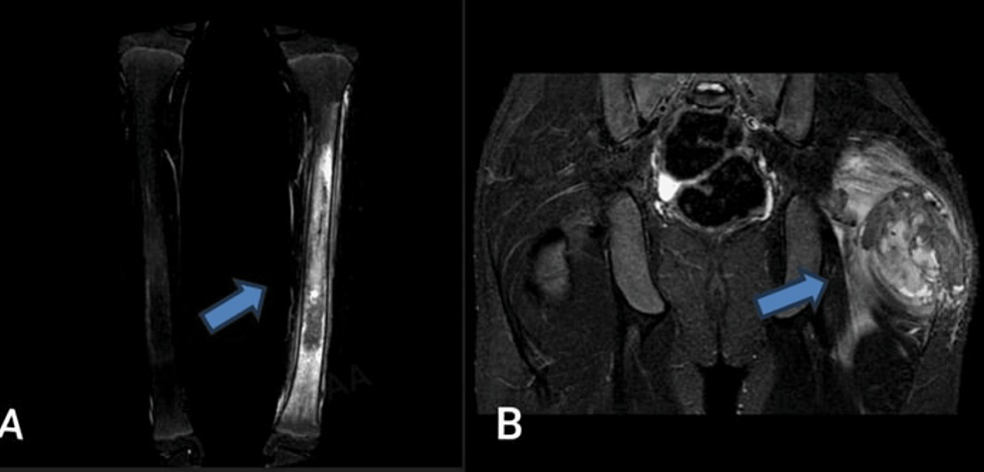
Case 1 revealed diffuse intra-medullary altered signal involvement of the left tibia (A) and a calcified mass in the left hip (B)

On follow-up at the age of 15 years, he developed a left gluteal mass. A pelvic MRI scan (Figure 1) showed a 12x9x6 cm mass in the left gluteal muscle with no bone involvement. A biopsy under ultrasound (US) guidance was performed, and the results indicated tumoral calcinosis. Therefore, the orthopedic team recommended that he undergo a complete excision of the mass. 

**Figure 2:**
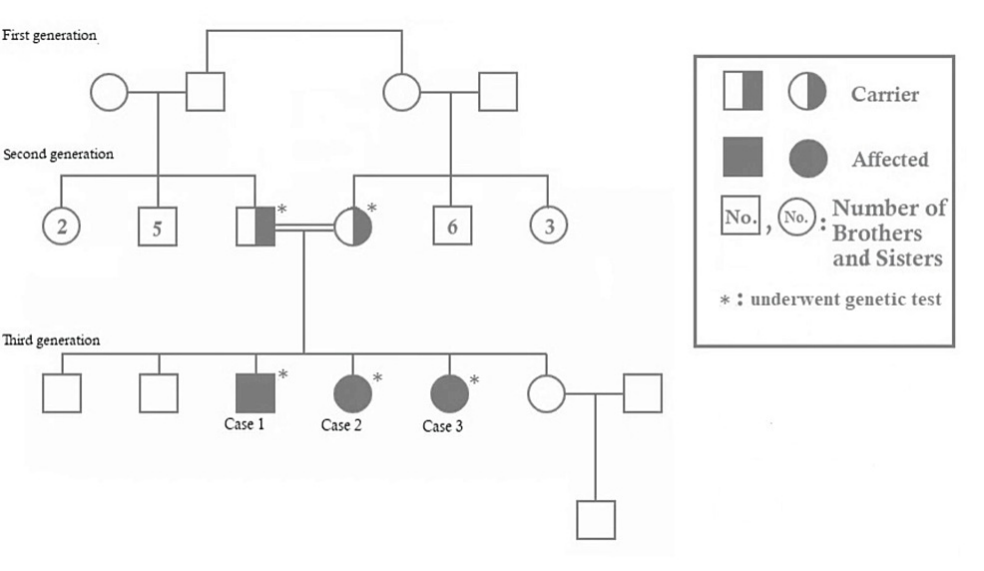
Family pedigree chart

When a family history was taken, the parents were found to be first cousins. Additionally, two sisters had similar complaints, so they were admitted later for further assessment and management. Segregation study and testing of other family members for this mutation were recommended. Segregation analysis study showed that parents are carriers of the same genetic mutation (see Figure 2).

Case 2

An 11-year-old female patient presented to our hospital complaining of right leg pain for one and a half years. She started having mild pain while walking and was incidentally discovered to have a lump over the lateral aspect of her right hip. There was no history of fever, weight loss, or trauma. The review of systems was unremarkable. 

On physical examination, the lump was 1x2.5x5 cm, hard and tender, with normal overlying skin color. It was mobile and not pulsatile. The remainder of the physical examination was normal.

**Figure 3:**
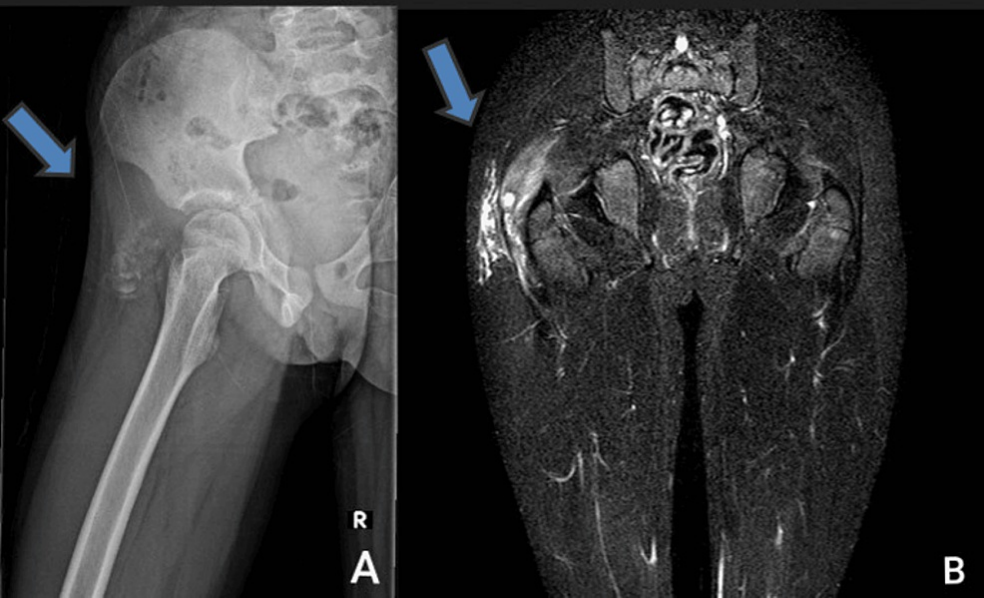
Case 2 X-ray revealed soft tissue calcifications around the right greater trochanter consistent with tumoral calcinosis (A) and MRI revealed a calcified right hip mass (B)

Blood work, described in Table 1, showed a high serum phosphate level (8.45 mg/dl). An X-ray of the pelvis (Figure 3A) and MRI (Figure 3B) revealed soft tissue calcifications around the right greater trochanter consistent with tumoral calcinosis. 

Genetic testing was sent for the same mutation in the GALNT3 gene that was detected in her brother. She was also found to be homozygous for mutation c.1524+1 G>A (IVS8+1) in intron eight of the GALNT3 gene. The patient was maintained on a low phosphorus diet. She was started on a phosphorus-chelating agent (aluminum hydroxide) to which she was not compliant and was given NSAIDs as needed. During her last follow-up, surgical excision of the lump was recommended as it was gradually getting bigger in size and causing her significant discomfort.

Case 3

A nine-year-old female patient was doing well until the age of six years when she started to complain of recurrent left-sided lower leg pain that was responsive to NSAIDs. She had no history of fever, and the review of systems was unremarkable. On physical examination at the age of nine years, she had localized tenderness over the mid-shaft of the left lower leg but no palpable masses.

**Figure 4:**
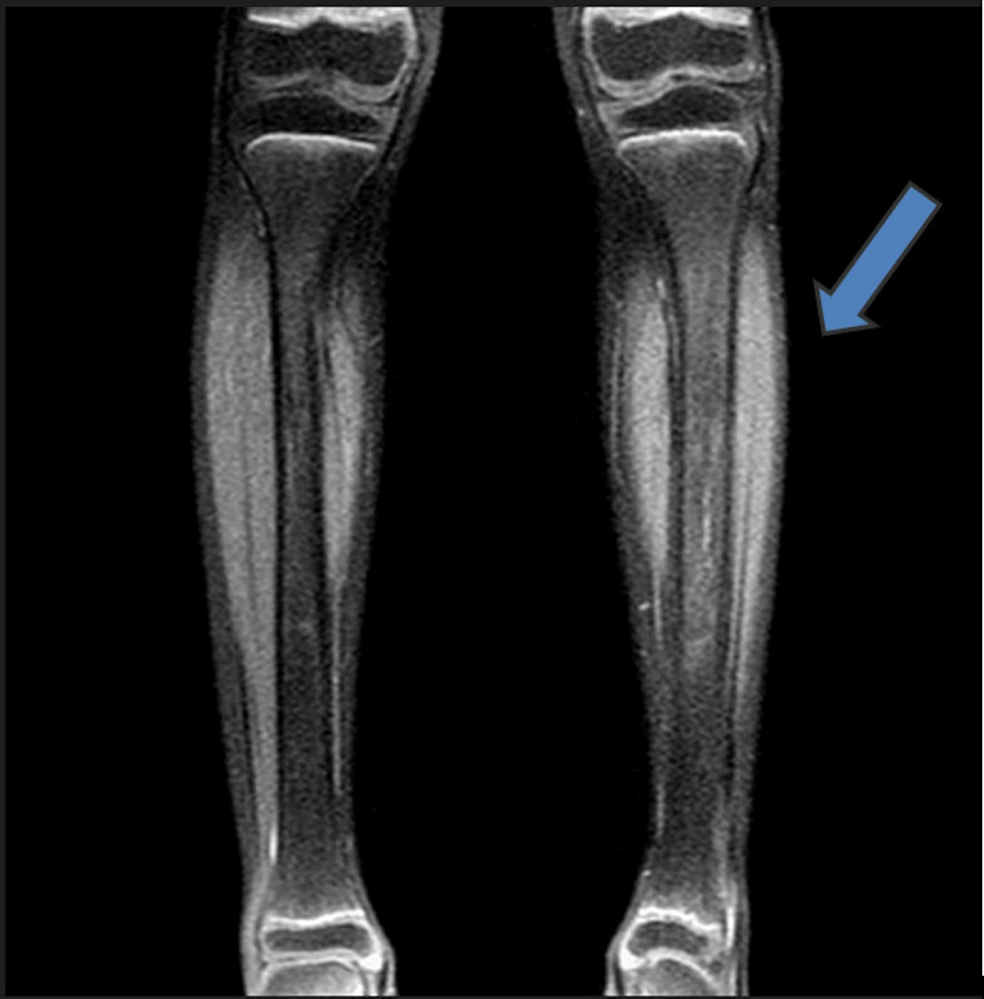
MRI revealed a patchy high stir signal in the mid-diaphysis of the left leg (tibia)

Blood work, described in Table 1, showed a high serum phosphate level (7.75 mg/dl). X-ray of the lower legs showed a periosteal reaction. MRI with contrast (Figure 4) showed a patchy high stir signal in the mid-diaphysis of the left leg with no effusion in the knees or ankles to be found. Genetic testing showed that she is homozygous for mutation c.1524+1 G>A (IVS8+1) in intron 8 of the GALNT3 gene.

The patient was also maintained on a low-phosphorus diet. She was given a phosphorus-cleating agent (aluminum hydroxide), to which she was also not compliant, and was given NSAIDS as needed.

## Discussion

HHS/HFTC are rare autosomal recessive diseases (Genetic and Rare Diseases Information Center, GARD 0010879); approximately 75 cases have been genetically described worldwide [8,10,11]. They are caused by mutations in the GALNT3, FGF23, and KL genes. Our patients were found to be homozygous for the mutation c.1524 G>A (IVS 8+1) in intron 8 of the GALNT3 gene. The GALNT3 enzyme initiates 0-glycosylation of FGF23 in a furin-like convertase recognition sequence and prevents proteolytic processing of FGF23, allowing the secretion of intact FGF23. This mutation of GALNT3 leads to the destabilization of FGF23 and makes it susceptible to proteolysis, resulting in the loss of FGF23 function [11].

Tumoral calcinosis is considered one of the most common manifestations of the disease; it usually occurs in the soft tissues around the joints, most often the hip joints, elbows, and shoulders. In rare cases, calcinosis occurs in the blood. It develops over time and varies in size; if large enough, it can interfere with the function of the joint and impair its movement, necessitating surgical removal [12]. Our first and second cases developed tumoral calcinosis close to the hip joint, and the first case underwent excision due to worsening symptoms in addition to its big size, while it was recommended later on follow-up for the second case for similar reasons. Dental and eye involvement have been reported to be associated with the disease, but none of our patients had eye or dental involvement. 

Many patients report bone pain, particularly in the tibia, with localized tenderness, redness, and warmth mimicking osteomyelitis. Our third patient presented with localized tibial tenderness as our first case, who was thought to have recurrent osteomyelitis.

All of our patients had hyperphosphatemia, which is thought to be secondary to increased renal tubular phosphate reabsorption. The workup of our patients showed normal renal function tests, parathyroid hormone(PTH), calcium levels, alkaline phosphatase, CBC, and inflammatory markers to exclude other causes.

Many complications may occur in HHS/HFTC, like ulceration of the skin, secondary infections, an increased risk of cardiac events, and sudden visual loss due to subretinal hemorrhage [10,13]. Management focuses on lowering serum phosphorus levels through a low-phosphorus diet and the use of phosphorus-chelating agents, in addition to decreasing the level of inflammatory markers. The medications currently prescribed for phosphorus reduction include lanthanum, sevelamer carbonate, and aluminum hydroxide, which are phosphate-binding agents that decrease phosphate intestinal absorption [14-16]. Acetazolamide, which causes proximal renal tubular acidosis and increases urinary phosphate excretion, can be used [17]. Other drugs like nicotinamide and niacinamide contribute by inhibiting the sodium phosphate co-transporter in the proximal renal tubules of the kidney and intestine, thus decreasing the urinary and intestinal absorption of phosphorus [18,19].

Our patients were maintained on a low-phosphorus diet. A dietician was involved in their management. However, they were not compliant with the oral phosphate binders. NSAIDs on demand were effective in the management of hyperostosis and the signs and symptoms of inflammation. Follow-up blood work, including CBC, inflammatory markers, renal function tests, calcium, magnesium, and serum lytes other than phosphorus, remained normal. Phosphorus levels were mildly reduced, which could be attributed to non-adherence with medical therapy, though the reported results of the use of phosphate-lowering drugs in this condition have not been consistent.

## Conclusions

This case series described three cases of HHS/HFTC in consanguineous Palestinian families who were found to have the same genetic mutation with combined phenotypes of both diseases. Being a rare disease, the findings of tumoral calcinosis and/or bony abnormalities, along with elevated phosphate levels, should raise the possibility of this entity, thus preventing unnecessary investigations or inappropriate management.
